# Multiple Organ Phenotype of Fatigue

**DOI:** 10.3390/biom15101476

**Published:** 2025-10-20

**Authors:** Xiaohua Liu, Zhonghan Zhao, Yuan Zhang, Jun Zou, Lingli Zhang

**Affiliations:** 1 School of Exercise and Health, Shanghai University of Sport, Shanghai 200438, China; 2511517008@sus.edu.cn (X.L.); 2221516004@sus.edu.cn (Z.Z.); junzou@sus.edu.cn (J.Z.); 2 College of Athletic Performance, Shanghai University of Sport, Shanghai 200438, China; 2221111032@sus.edu.cn

**Keywords:** fatigue, multiple organs, phenotype, bone, muscle

## Abstract

Fatigue is not only a widespread subjective experience but also a complex physiological and pathological state involving multiple organs and systems. Currently, there is no consensus on the definition and classification of fatigue. Based on its causes, this paper categorizes fatigue into sports fatigue, occupational fatigue, and pathological fatigue. It elaborates on the specific manifestations and underlying mechanisms of fatigue in the motor, nervous, cardiovascular, digestive, urinary, endocrine, and reproductive systems, aiming to uncover the intrinsic connections of fatigue phenotypes across different systems. These findings may provide key targets for gene-assisted therapy of fatigue-related complications, thereby establishing a new theoretical foundation for the clinical management of fatigue and related research.

## 1. Introduction

Fatigue is a complex subjective sensation, and there is currently no globally unified definition or consensus. The concept of fatigue can be traced back to the industrial revolution era as early as 1880. The Italian physiologist Angelo Mosso observed that most workers, including children, who were exposed to prolonged physical labor in harsh industrial environments, faced significant health risks. This discovery laid the groundwork for subsequent investigations into fatigue. In 1915, Mosso proposed that fatigue results from the intoxicating effects of cellular chemical changes. Later, in 1979, Karpovich argued that fatigue is a decline in work capacity induced by the work itself [1]. Since then, numerous scholars have explored the mechanisms of fatigue from diverse perspectives using a variety of methodologies [2,3]. In the medical field, fatigue generally refers to a diminished capacity for work caused by overexertion, whether physical or mental. It is characterized by slowed reactions, decreased agility and coordination, increased work errors, and accompanied by subjective sensations of fatigue and weakness. In essence, fatigue represents an intermediate functional state of the body, situated between the extremes of alertness and sleep. It arises from a multitude of physiological changes and can even lead to physiological dysfunctions or disorders. Currently, scholars from diverse disciplines lack a unified understanding of the mechanisms underlying fatigue, resulting in contentious divisions within the field.

Research indicates that although fatigue can be broadly divided into physiological and pathological types [4], a universally accepted classification system is still lacking. Through a comprehensive literature review, several causes of fatigue have been identified and can be broadly categorized into three main types. Firstly, sports fatigue arises from excessive training volume, training load, training frequency, and training density [5,6]. Secondly, occupational fatigue occurs as a result of prolonged maintenance of certain postures or enduring high-pressure conditions, commonly observed in professionals such as pilots, drivers, engineers, experimenters, and medical personnel. Finally, pathological fatigue emerges during the recovery period before and after specific diseases or surgical procedures [5,6].

In summary, we propose that fatigue represents a comprehensive manifestation of multisystem compensatory dysfunction in response to persistent internal (e.g., disease, inflammation) or external (e.g., stress, overtraining) stressors. This article systematically describes the impact and underlying mechanisms of exercise-induced, occupational, and pathological fatigue on various organ systems, aiming to provide a theoretical basis for future fatigue treatment.

## 2. Multiple Organ Phenotype Induced by Fatigue

Long-term fatigue that cannot be fully alleviated by rest is often accompanied by coordinated or independent functional impairments across multiple organ systems, primarily affecting the skeletal muscle, bone, brain nerve, heart, kidney, and other organs.

### 2.1. Influence of Fatigue on the Locomotor System

#### 2.1.1. Influence of Fatigue on Muscle

The locomotor system is an organic whole composed of skeletal muscles, bones, and joints, where skeletal muscles are not only the executors of exercise, but also the key factors in maintaining body function, metabolic health, and overall physiological balance. After prolonged high-intensity exercise, fatigue is highly likely to occur and manifest as impaired muscle function. Muscle fatigue prevents the maintenance of the necessary strength for a specific task or the generation of expected strength [7,8]. It alters the performance of evident muscle contractions, leading to sluggish and awkward task execution, and in some cases, an inability to achieve success. Moreover, it can disrupt the neuromuscular activity necessary for task completion, resulting in changes in electromyography readings. Additionally, muscle fatigue can cause pain or discomfort. The diverse causes of muscle fatigue give rise to distinct physiological mechanisms underlying each phenomenon.

Sports fatigue is primarily characterized by muscle fatigue, which refers to a decrease in muscle or muscle group capacity to produce or sustain strength due to exercise, thereby limiting exercise performance and duration. This type of fatigue can manifest immediately after exercise (acute muscle fatigue), which can be quickly alleviated through rest or lifestyle changes. Alternatively, it can occur following long-term, high-intensity exercise (delayed sports fatigue) and last for months without relief from rest [9]. The reduction in muscle strength during exercise can be seen as a protective mechanism. If fatigue did not occur or was delayed, muscle cell function decline and structural damage to muscle fibers would be more likely to occur during exercise [9]. Acute muscle fatigue is primarily characterized by metabolic disturbances and energy depletion. During exercise, muscles undergo anaerobic respiration, producing lactate, inorganic phosphate, Ca^2+^, Mg^2+^, and other metabolites. The accumulation of these substances alters the intramuscular pH, thereby impairing muscle function. Previous studies have confirmed that sports fatigue leads to significant lactic acid accumulation in muscles [10]. The breakdown of lactic acid results in excessive hydrogen ion (H^+^) and carbon dioxide metabolism, directly lowering the intramuscular pH and creating an acidic environment [11]. This, in turn, impairs the ability of the sarcoplasmic reticulum to absorb Ca^2+^ and increases the cytoplasmic Ca^2+^ concentration, ultimately compromising skeletal muscle strength [12]. Furthermore, hydrogen ions released from lactic acid compete with Ca^2+^ for binding sites on troponin, reducing troponin’s sensitivity to Ca^2+^ and disrupting the interaction between thick and thin myofilaments [12], eventually leading to a loss of muscle contractility. Adenosine triphosphate (ATP) serves as the energy source for muscle contraction. When sports fatigue occurs, muscular energy metabolism is disrupted, hindering the continuous resynthesis of ATP in muscle cells and impairing the number or function of actin and myosin cross-bridges.

Delayed-onset muscle fatigue is mainly manifested as microstructural damage and oxidative stress. Compared to other types of muscle actions, eccentric contractions induce microdamage with higher frequency and severity, disrupting the normal alignment of muscle fibers [13,14]. Moreover, the production of free radicals during exercise is related to the strength of muscle contraction. Most studies suggest that ROS-induced phagocyte infiltration and the subsequent inflammatory response contribute partially to delayed-onset muscle damage following prolonged exercise [15,16]. Sports fatigue triggers a notable rise in ROS, leading to mitochondrial dam-age in skeletal muscles and disrupting the redox balance of working muscles, thereby impairing muscle performance [17]. In the resting state, an appropriate amount of free radicals can promote vasodilation, while the production of free radicals increases significantly during exercise, but excessive free radical levels will inhibit vasodilation and reduce blood flow, causing insufficient supply of oxygen and nutrients required by various tissues and organs of the body such as skeletal muscle, heart, and brain, and accelerating the production of fatigue [18]. Sports fatigue alters electromyography imaging of skeletal muscles. When muscle fatigue sets in, the integrated EMG amplitude and root mean square amplitude increase, while the mean power frequency and median frequency decrease [19,20]. Finally, Long-term high-intensity sports fatigue can promote the expression levels of pro-inflammatory cytokines (IL-1β, IL-6, and TNF-α), accompanied by muscle damage [21]. Studies have shown that elevated levels of inflammatory factors can activate protein degradation pathways, inhibit protein synthesis, and impair the function of muscle stem cells, leading to muscle atrophy [22].

The impact of occupational fatigue on muscles is closely linked to the nature of the occupation. Pilots, for instance, require sustained focus and a fixed driving posture while flying. This prolonged static isometric contraction of the shoulder, neck, back, and lower limb muscles contributes to muscle fatigue in pilots. The static nature of their work exacerbates the load on muscle groups, depriving them of intermittent rest and reducing muscle blood flow, thereby overburdening the working muscles [23,24]. This occupational fatigue commonly leads to stiffness and pain in the neck, back, and other muscles of pilots. Previous studies have demonstrated that healthcare professionals experience occupational fatigue primarily due to the prolonged maintenance of a stooping and bending posture. This leads to spinal misalignment, increased cervical and lumbar spine load, continuous tension on the neck, shoulder, and back muscles, muscle group imbalances, compromised blood circulation, and muscle strain issues [25,26,27].

Pathological fatigue can disrupt muscle metabolism, cause mitochondrial damage, decrease ATP synthesis, and ultimately result in muscle dysfunction, heightened inflammation, and significant pain [28]. For instance, cancer-related fatigue can induce metabolic disturbances in muscles. Cancer or its treatment can damage the sarcoplasmic reticulum, increase intracellular calcium levels, impair mitochondria, and disrupt muscle metabolism. Sarcoplasmic reticulum damage can lead to the buildup of metabolites at the neuromuscular junction and within the muscles, as well as reduce the sensitivity of actin and myosin to calcium or ATP by interfering with Ca^2+^ release from the sarcoplasmic reticulum, thereby causing fatigue [29,30,31]. Furthermore, ATP serves as the primary source of energy for skeletal muscle contraction. Under normal circumstances, ATP can be rapidly replenished. However, changes in appetite and treatment side effects in cancer patients often result in reduced energy intake, subsequently affecting ATP production [32]. As a result, the body cannot adequately replenish ATP, leading to impaired muscle function and reduced physical labor capacity.

Overall, muscle fatigue is characterized by reduced muscle strength, metabolic disruptions, and pronounced pain.

#### 2.1.2. Influence of Fatigue on Bone

Bones are dynamic and active organs within the body that serve functions such as support, movement, protection, and hematopoiesis. Physical activity improves overall health by increasing bone density, improving muscle growth and strength, the cardiovascular system, and reducing body fat. However, strenuous and excessive exercise may overshoot the body’s ability to recover, compromising bone health [33]. Fatigue is a typical response of materials subjected to cyclic loading of a specific intensity, characterized by the formation of micro-cracks. Bone tissue, being the primary weight-bearing structure, is not exempt from this phenomenon. Bone fatigue commonly occurs in activities such as long-distance running and military recruit training [34]. When a sufficiently large load is applied to the bone, it undergoes deformation or strain. If the strain reaches a high level, it can damage the microstructure of the bone tissue. Progressive loading and reduction in bone hardness increase bone strain, resulting in further accumulation of micro-damage, impaired repair processes, and eventual occurrence of stress fractures [35]. Research indicates that the incidence of stress fractures is age-related. A study involving 2312 active-duty Army women revealed that the prevalence of stress fractures was as high as 19.6% in women aged 22–23, but only 1.4% in women over 40 [36]. Fyhrie et al. [37] attributed this phenomenon to decreased muscle coordination and an increased bone strain rate in young individuals, leading to a higher incidence of stress fractures.

Bone metabolism includes bone formation and bone resorption, which is the process of interaction between osteoblasts and osteoclasts. According to the study by Barbe et al. [38], rats performing long-term high-load and high-repetition tasks developed fatigue, which led to significant decreases in bone volume fraction (BV/TV) and trabecular number (Tb.N), along with significant increases in trabecular separation (Tb.Sp) and anisotropy in the distal radius. Bone formation was impaired, as indicated by a marked reduction in bone formation rate (BFR), accompanied by upregulation of SOST and a decrease in osteoblasts. Furthermore, the release of RANKL promoted osteoclast activity, enhancing bone resorption. SOST, which encodes sclerostin, has been shown to inhibit bone formation by specifically binding to the Wnt co-receptor LRP5, thereby suppressing Wnt signaling [39]. Meanwhile, RANKL is closely associated with osteoclast-mediated bone resorption. These findings suggest that sports fatigue may alter the expression of genes and proteins related to both bone formation and resorption, thereby disrupting bone remodeling and compromising skeletal health. Another study observed that after completing a 250 km ultramarathon, athletes exhibited a significant increase in serum CTX-I and a notable decrease in osteocalcin (OCN). Three days post-race, RANKL levels were further elevated, while osteoprotegerin (OPG) showed a non-significant decrease [40]. These results indicate that sports fatigue suppresses bone formation and promotes bone resorption, which is consistent with the findings of Mouzopoulos et al. [41]. They suggested that the reduction in bone formation markers may be related to inhibited osteoblast function, and also noted increased release of cortisol and PTH due to sports fatigue.

Therefore, in a long-term state of fatigue, bone fatigue may occur, and in severe cases, it can lead to the occurrence of fractures; In addition, it can also affect bone metabolism by reducing bone formation and promoting bone resorption, leading to loss of bone mass.

#### 2.1.3. Influence of Fatigue on Joint

**Figure 1:**
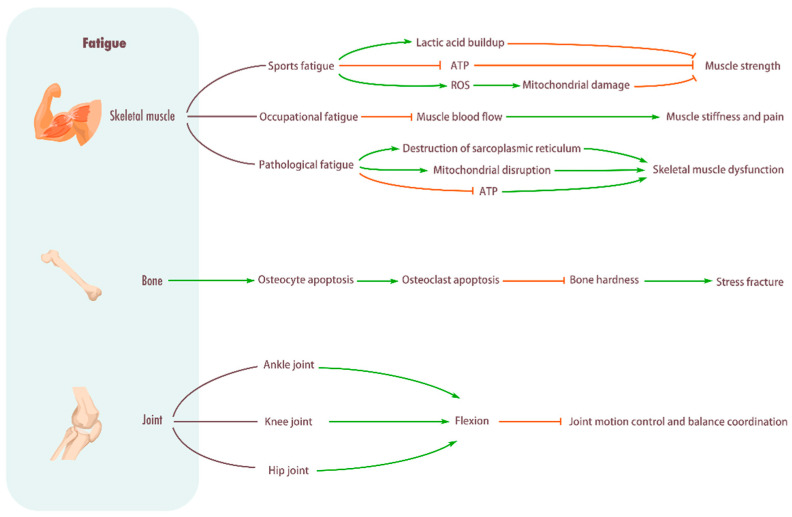
Influence of fatigue on musculoskeletal system. Skeletal muscle: Sports fatigue causes a significant buildup of lactic acid in the muscles, hinders ATP production, promotes ROS, damages mitochondria, and reduces muscle strength. Occupational fatigue can impede muscle blood flow and cause stiffness and pain in muscles. Pathological fatigue can damage the sarcoplasmic reticulum, damage mitochondria, impede ATP production, and cause skeletal muscle metabolic disorders. Bone: Promotes osteocyte apoptosis, increases osteoclasts, decreases bone stiffness, and causes stress fractures. Joint: Fatigue will lead to pelvic forward tilt, increased flexion of hip and knee joints, and increased dorsiflexion of ankle joints, thus reducing the ability of motion control and balance coordination.

When the body is tired, it can lead to varying degrees of decline in muscle strength, joint movement control, and balance and coordination. While the effects of sports fatigue on muscles and bones have been extensively studied, less research has been done on how sports fatigue affects joints and thus alters exercise mechanics and energetics. Wang et al. [42] showed that weight-bearing and fatigue walking led to significant changes in lower limb joint mechanics, with weight-bearing walking leading to increased hip and knee flexion with anterior pelvic tilt and heel contact, and fatigue walking leading to increased ankle dorsiflexion when heel contact. Another study found that sports fatigue was followed by smaller plantar flexion, knee flexion, and hip flexion angles, as well as larger dorsiflexion angles, and fatigue did not alter joint torque, but power expenditure was redistributed, with ankle and knee joints reduced but hip joints increased [43]. Kao et al. [44] found that the energy expenditure of healthy people increased significantly when walking at the same speed after sports fatigue, and the ability of the ankle joint to generate positive mechanical power was most affected by walking-induced sports fatigue, and the reduction in ankle kick-off may be compensated for by the increase in mechanical power generated by the knee joint, resulting in higher metabolic costs during walking after sports fatigue. Fatigue induced by running leads to significant alterations in lower limb biomechanics, particularly in kinetic parameters. Specifically, the hip joint demonstrates a decrease in peak flexion moment, along with increases in peak adduction and abduction moments, peak external rotation power, and peak adduction and abduction power. At the knee joint, increases are observed in peak abduction and external rotation moments, peak flexion power, and peak adduction and abduction power. For the ankle joint, elevations are noted in peak external rotation moment, peak abduction power, and peak internal rotation power. These fatigue-induced compensatory adaptations across multiple joints substantially increase the risk of sports-related injuries [45]. In conclusion, exercise-induced fatigue can affect the motion mechanics of lower limb joints, that is, the reduction in joint motion control and balance coordination (Figure 1).

### 2.2. Influence of Fatigue on the Nervous System

#### 2.2.1. Influence of Fatigue on the Central Nervous System

Sports fatigue can have a significant impact on the central nervous system, leading to the occurrence of central fatigue. Central fatigue primarily arises from the cerebral cortex to the motor neurons in the spinal cord. Sports fatigue affecting the central nervous system inevitably results in reduced excitability, decreased nerve impulses, and diminished muscle activity [10]. Firstly, sports fatigue markedly reduces ATP levels in cerebral cortex cells, increases adenosine diphosphate (ADP) concentration, elevates the ADP/ATP ratio, and decreases blood glucose levels. It also leads to a significant rise in gamma-aminobutyric acid, brainstem and hypothalamic serotonin (5-hydroxytryptamine) concentrations, increased brain ammonia content, and reduced succinate dehydrogenase activity. These phenomena reduce ATP synthesis rate in brain cells, lower the excitability of cerebral cortical cells, and diminish nerve impulse frequency, thereby compromising the central nervous system’s regulatory capacity [14]. Moreover, the hypothalamus and hippocampus are vital components of the brain’s limbic system. When subjected to the stress of trauma, illness, or environmental changes, they become particularly vulnerable, resulting in alterations in mood, behavior, and higher-level cognitive functions [46]. Studies have shown that sports fatigue can induce morphological changes such as astrocyte (AS) cell hypertrophy and dendritic thickening in the hippocampus of rats, along with increased expression of glial fibrillary acidic protein (GFAP), thus intensifying hippocampal damage and impairing learning and memory function [47]. Furthermore, sports fatigue can inhibit the transmission of nerve impulses from the spinal cord’s alpha motor neurons, causing changes in central nervous system excitability and reduced signals being relayed to the muscle nervous system, ultimately leading to a decline in skeletal muscle performance [48,49]. Research has shown that the impact of sports fatigue on the central nervous system may be related to neuroinflammation [50]. In summary, the adverse effects of sports fatigue on the central nervous system exacerbate bodily fatigue and compromise exercise capacity.

Occupational fatigue also adversely affects the central nervous system, but it impacts different regions and can easily lead to decreased job performance. For example, fatigue diminishes pilots’ alertness and acuity, and the high mental workload experienced during flights can also impair their flight performance [51]. Increased workload during flight missions is strongly associated with a general decrease in frontal brain regions’ EEG beta, theta, and alpha frequencies. Simultaneously, it coincides with increased oxygenated blood (HbO_2_) and decreased deoxygenated blood (hHB) levels in pilots’ prefrontal cortex, leading to cognitive decline and mental fatigue in fatigued states [52,53]. Moreover, occupational fatigue can impose overwhelming stress on healthcare workers, resulting in increased release of norepinephrine and dopamine, which negatively impact the prefrontal cortex responsible for top-down regulation of thoughts, actions, and emotions. Consequently, prefrontal cortex dysfunction adversely affects the cognitive ability, decision-making skills, insight, and adaptability of medical professionals, leading to job burnout and exacerbating the doctor-patient relationship strain [54]. It is evident that the impact of occupational fatigue on the central nervous system should not be underestimated. Therefore, both sports fatigue and occupational fatigue adversely affect the central nervous system, but their mechanisms of action differ. The impact of sports fatigue on the central nervous system is primarily characterized by an imbalance in energy metabolism, mainly affecting brain regions involved in motor control and regulation, such as the hypothalamus and hippocampus, which are associated with learning and memory. In contrast, occupational fatigue manifests more as sustained psychological stress and emotional changes, primarily impacting brain regions responsible for cognition and emotional regulation, like the prefrontal cortex. In the future, elucidating the differences between sports and occupational fatigue at the level of central nervous system mechanisms will provide a precise scientific basis for subsequent clinical treatment and intervention.

Pathological fatigue affects the central nervous system and is contingent upon the specific type of disease. Fatigue associated with cancer may be related to the basal ganglia and suprachiasmatic nucleus [55], often accompanied by increased activity in the basal ganglia and cerebellum. The suprachiasmatic nucleus, a component of the hypothalamus, plays a significant role in regulating circadian rhythms. Alterations in the corticosterone curve tend to be gradual, leading to an imbalance in suprachiasmatic nucleus levels that further exacerbates cancer-related fatigue [56]. Fatigue is one of the most common and highly disabling symptoms in Parkinson’s disease, with a distinct pattern that differs from fatigue in other conditions. Studies have shown a specific and strong association between fatigue and visuospatial dysfunction. This relationship may be explained by shared mechanisms such as insular dysfunction, in addition to the fact that visuospatial tasks require increased cognitive effort from patients, which may directly contribute to the experience of fatigue [57]. Positron emission computed tomography (PET) scans of 12 patients with Parkinson’s fatigue revealed increased glucose metabolism in regions such as the insula and posterior cingulate gyrus [58]. Furthermore, observations using single-photon emission computed tomography (SPECT) showed decreased frontal lobe perfusion and reduced binding of D2 receptors in the bilateral insula among patients with Parkinson’s fatigue, resulting in diminished executive and cognitive function [59]. These findings emphasize the importance of considering the impact of pathological fatigue on the central nervous system during the evaluation of fatigue processes.

#### 2.2.2. Influence of Fatigue on the Peripheral Nervous System

**Figure 2:**
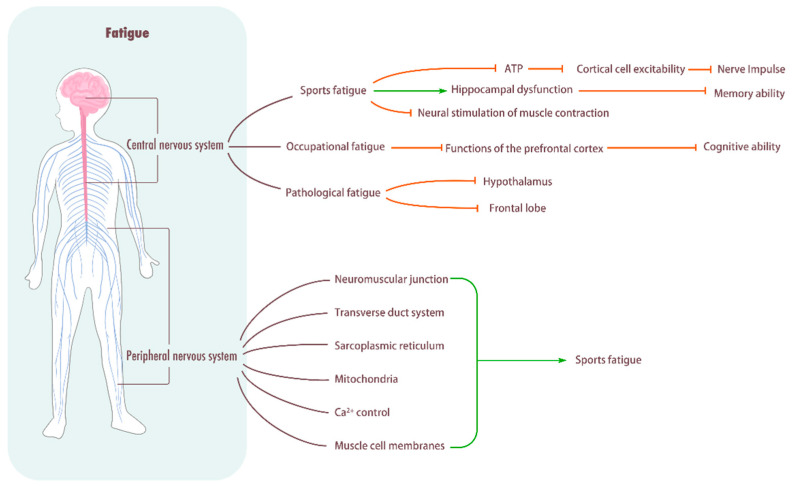
Influence of fatigue on central system. Central nervous system: Sports fatigue decreases the rate of ATP synthesis in brain cells, reduces the excitability of cortical cells, and hinders the frequency of nerve impulse delivery. In addition, it causes hippocampal dysfunction, decreases memory ability, and impedes neural stimulation of muscle contraction. Pathological fatigue can damage the hypothalamus and frontal lobe. Peripheral fatigue: Peripheral fatigue occurs in the neuromuscular junction, transverse tube system, sarcoplasmic reticulum, mitochondria, Ca^2+^ control, and muscle cell membrane, and it easily leads to sports fatigue.

Sports fatigue can also have a significant effect on peripheral nerves, causing peripheral fatigue. Peripheral fatigue occurs at the neuromuscular junction, transverse duct system, sarcoplasmic reticulum, mitochondria, Ca^2+^ control, and muscle cell membranes. Thomas et al. [60] investigated the task-dependency of neuromuscular fatigue by having 13 male cyclists complete 4 km, 20 km, and 40 km cycling time trials, with relevant neuromuscular indicators assessed before and immediately after exercise. The results demonstrated that after the high-intensity 4 km trial, the knee extensors exhibited significant reductions in maximal voluntary contraction force, potentiated twitch amplitude, maximal rate of force development, and one-half relaxation time, indicating that fatigue was predominantly peripheral in origin. In contrast, following the longer-duration trials, central fatigue became more prominent, which was primarily manifested as a reduction in corticospinal excitability. During prolonged exercise, Ca^2+^ delivered to the sarcoplasmic reticular tissue decreases, resulting in reduced energy production and inability to meet exercise needs, resulting in sports fatigue [61]. Further research is needed on the effects of sports fatigue on the peripheral nerves (Figure 2).

### 2.3. Influence of Fatigue on the Cardiovascular System

#### 2.3.1. Influence of Fatigue on Heart

In the cardiovascular system, sports fatigue has a more significant impact on the heart. Intense exercise can temporarily elevate the risk of acute cardiac events in athletes, with endurance athletes being susceptible to atrial fibrillation. Engaging in marathon exercise poses the risk of transient cardiovascular changes and permanent remodeling [62]. During high-load and high-intensity exercise, as the body progresses, the heart’s blood pumping volume increases while consuming substantial energy resources. As exercise intensity further intensifies, heart rate accelerates and the period of cardiac relaxation shortens. Consequently, the filling time for blood in the heart diminishes, leading to inadequate blood supply in the coronary artery and ultimately causing myocardial ischemia and hypoxia [63]. When myocardial homeostasis deviates significantly from the normal threshold range, its inherent regulatory mechanisms become insufficient to maintain balance, resulting in structural damage to the myocardium. Intense exercise-induced damage is often irreversible [64,65]. Kadaja et al. [66] discovered that 6 weeks of treadmill training, inducing chronic physical exertion (overtraining), led to the collapse of the rat myocardium’s myofibrillar structure, peroxisome generation, and cell swelling. Concurrently, the efficiency of oxidative phosphorylation in rat myocardial mitochondria decreased, resulting in myocardial damage and disruption in the production of ATP through mitochondrial oxidative phosphorylation, thus altering cellular energy metabolism. Furthermore, overtrained mice exhibited pathological left ventricular hypertrophy and cardiac fibrosis [67]. Sports fatigue heightens the risk of pathological cardiac remodeling, and certain key miRNAs (e.g., miR-1, miR-133a, miR-133b) in the left ventricle are upregulated due to overtraining, consequently affecting cardiac adaptability and remodeling [68]. Notably, training load significantly diminishes heart rate variability (HRV). HRV serves as a monitoring tool for training load optimization, aiding in the prevention of long-term athlete fatigue [69]. For instance, a sharp increase in training volume or sustained high-level training for 3–5 consecutive days results in a significant decrease in HRV, indicating excessive fatigue and inadequate recovery among athletes [70]. In conclusion, sports fatigue disrupts myocardial mitochondrial phosphorylation, leads to mitochondrial energy metabolism disorders, impacts left ventricular miRNAs, causes myocardial injury, and reduces cardiac function.

#### 2.3.2. Influence of Fatigue on Blood Vessels

Cardiovascular disease is an inseparable system, and fatigue can adversely affect blood vessels when it affects the heart. The reactive hyperemia index (RHI) is one of the high risks of mortality in patients with cardiovascular disease, and chronic fatigue and sleep deprivation are highly susceptible to cardiovascular events. Studies have shown that aging patients and those with heart failure with reduced ejection fraction exhibit exercise intolerance, premature fatigue due to impaired muscle metabolism, and limitations in oxygen transport and utilization. These symptoms are associated with declined vascular function [71]. Sports fatigue can cause hemodynamic changes that limit blood flow and alter steady-state arteriovenous oxygen differences, posing a cardiovascular threat [72,73]. The accumulation of chronic fatigue increases the incidence of cardiovascular disease, causes vascular damage, and increases the levels of inflammatory cytokines such as IL-6 and TNF-α [74]. Fatigue can also induce endothelial dysfunction, leading to impaired vasodilation and frequent cardiovascular disease [75,76]. In conclusion, the effects of fatigue on blood vessels are often associated with cardiovascular disease.

### 2.4. Influence of Fatigue on the Respiratory System

The effects of sports fatigue on the respiratory system are significant. During high-intensity aerobic or anaerobic exercise, the body needs more oxygen to meet the demands of muscle activity. This will increase the intensity of the lungs and respiratory muscles to increase oxygen intake and carbon dioxide excretion [77]. As you exercise, your breathing rate and depth of breathing gradually increase to meet the demands of your muscles. However, prolonged periods of high-intensity exercise can cause fatigue in the lungs, causing dyspnea or lung discomfort [78]. In addition, excessive exercise can lead to airway inflammation and airway spasm, further affecting respiratory function [79].

**Figure 3:**
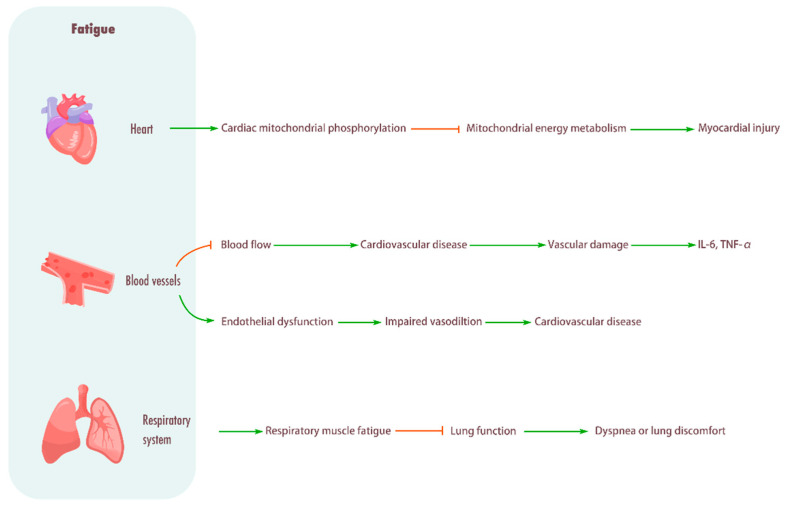
Influence of fatigue on cardiovascular system and respiratory system. Heart: Promotes cardiac mitochondrial phosphorylation, impairing mitochondrial energy metabolism and causing myocardial injury. Blood vessel: Fatigue will cause limited blood flow, increase the incidence of cardiovascular diseases, cause vascular injury, and enhance the levels of inflammatory cytokines IL-6 and TNF-α. Fatigue can also induce endothelial dysfunction, resulting in impaired vasodilation and frequent cardiovascular diseases. Respiratory system: Fatigue can cause respiratory muscle fatigue, lead to decreased lung function, and cause dyspnea or lung discomfort.

Research indicates that the ventilatory demands of prolonged endurance exercise can compromise resting pulmonary function and attenuate the functional work capacity of the respiratory muscles, which may, in turn, affect both health and endurance performance [80]. In a 107 km mountain ultramarathon, participants exhibited reductions in forced vital capacity, forced expiratory volume in 1 s, peak inspiratory flow, and maximal inspiratory pressure. Concurrently, significant elevations in creatine kinase and C-reactive protein were observed, indicating the occurrence of systemic fatigue [81]. Systemic sclerosis (SSc) is a disease associated with abnormalities in the lungs. Fatigue is a very common symptom in patients with SSc, and when fatigue increases, respiratory function is severely suppressed, followed by dyspnea, decreased peripheral and respiratory muscle strength, and deterioration of functional capacity [82]. In addition, fatigue can induce an increase in subjective fatigue in patients with chronic obstructive pulmonary disease (COPD), worsening the severity of the disease and further reducing the quality of life [83,84] (Figure 3).

### 2.5. Influence of Fatigue on the Digestive System

Exercise-induced fatigue can also affect digestive system function. During exercise, blood is preferentially distributed to working muscle groups, which reduces the blood supply to the digestive system, leading to a weakening of digestive function, causing gastric-abdominal discomfort or dyspepsia [85]. In addition, prolonged, high-intensity exercise can lead to dehydration and electrolyte imbalances. Dehydration causes dryness of the mucous membranes and decreased gastric acid secretion, which affects the digestion and absorption of food [86,87]. For example, Jakobsson et al. [88] found that fatigue can lead to intestinal damage, diarrhea, and even exacerbate depression and anxiety in inflammatory bowel disease (IBD).

Fatigue is an important component of chronic liver disease (CLD) and the most common complaint in the patient population [89,90]. Several studies have confirmed that fatigue can have a range of adverse effects in patients with liver disease. For example, fatigue can cause sleep disruption in patients with non-alcoholic steatohepatitis (NASH) and chronic hepatitis C (CHC), leading to mood changes consistent with anxiety, depressive symptoms, and decreased quality of life, exacerbating disease progression [91,92]. In particular, fatigue is more common in patients with end-stage of liver disease (ESLD) and predisposes to symptoms such as muscle wasting, malnutrition, and weakness, which can severely affect daily activities and quality of life [93]. In addition, serum creatine kinases (CK) are important kinases that are closely related to energy supply. Studies have demonstrated that patients with chronic fatigue consistently exhibit low CK levels [94], and that a decrease in CK synthesis ultimately disrupts ATP synthesis, affecting muscle function and overall health.

### 2.6. Influence of Fatigue on the Urinary System

The kidney is a commonly affected organ in sports injuries. During excessive exercise, blood perfusion increases in muscles, heart, and lungs, while the perfusion to the kidneys significantly decreases, leading to hypoxic injury of kidney tissue. High-intensity endurance runners experience increased Serum Creatinine (SCr) levels and changes in renal filtration, resulting in an elevated risk of acute kidney injury and impaired kidney function and structure [95]. Prolonged strenuous exercise primarily causes ischemic tubular injury due to reduced renal blood flow [96]. Lin et al. [97] observed that 24 h after exercise, rats experienced acute kidney injury characterized by increased blood urea nitrogen levels, damage to glomerular and tubular epithelial cells, increased apoptosis, and tubular dilation [98]. Sports fatigue leads to kidney damage, decreased glomerular filtration rate, reduced tubular reabsorption, and increased urinary protein content. Hematuria is a common manifestation of kidney injury, which may occur when the body has not fully recovered from fatigue after exercise [99]. Current studies mostly link kidney injury to exercise, and rarely explore the effects of fatigue on the kidneys. It is certain that kidney injury accompanies sports fatigue and may manifest as albuminuria or hematuria.

Urine testing of patients with CFS reveals lower levels of free cortisol in the urine than normal [100]. The investigators believe that this may be related to the decrease in adrenal output, and further speculate that the occurrence of CFS is related to the dysfunction of the adrenal system. Patients with non-metastatic prostate cancer often experience persistent fatigue, which is often clinically manifested as urinary dysfunction. Persistent fatigue caused by prostate cancer may be associated with a range of symptoms of chronic inflammation [101]. Due to the fact that there are currently few studies on the effects of fatigue on the urinary system. Therefore, the relationship between fatigue and the urinary system still needs further in-depth research.

### 2.7. Influence of Fatigue on the Endocrine System

Overtraining syndrome (OTS) is described as a form of chronic fatigue and burnout due to an imbalance between training/competition and recovery [102]. This exercise-induced fatigue can upset the balance of the endocrine system, which in turn affects the function of the adrenal glands and gonads.

First, exercise can affect the size and function of the adrenal glands. The adrenal gland size is increased by 31.04% in the mice with high-intensity exercise, and by 10.08% in the low-intensity exercise mice, and the adrenal medulla is significantly increased with high intensity [103]. A certain intensity of exercise is beneficial for adrenal function, but when exercise is performed excessively, sports fatigue occurs, and OST occurs. Adrenocorticotropic hormone (ACTH) and cortisol are two essential hormones required to maintain performance and speed during any exercise [104]. Corticotropin-releasing hormone (CRH) overactivity is thought to be associated with overtraining syndrome. Overtrained long-distance runners have a significantly reduced pituitary ACTH response, reflecting reduced hypothalamic and/or pituitary responsiveness and adrenal responsiveness to adrenocorticotropic hormone [105]. Cortisol, a hormone secreted by cortisol-producing cells in the adrenal cortex, is also affected. In overtrained long-distance runners, the maximum exercise-related cortisol levels decreased compared with baseline [106,107]. In addition, the OTS group had lower cortisol levels during and 30 min after hypoglycemia compared to the exercise-healthy group, and the mean cortisol rose slowly. In the insulin tolerance test, the ACTH/cortisol ratio was significantly lower in the OTS group compared with the healthy inactive group. These suggest that athletes affected by OTS have intrinsic dysfunction of the hypothalamic–pituitary–adrenal gland (HPA) in stressful situations [104]. In addition, patients with chronic fatigue syndrome have decreased adrenal secretion of cortisol, including basal cortisol and post-activity cortisol [108].

The effect of excessive exercise on the gonads is also manifested in the secretion of related hormones. Long-term strenuous running exercise (80% VO_2max_, 2 mice per exercise, 5 times/week for one year) resulted in significant reductions in plasma luteinizing hormone, follicle-stimulating hormone, and testosterone concentrations. Low serum levels of follicle-stimulating hormone, luteinizing hormone, and testosterone suggest hypothalamic-pituitary disorders, hypogonadism) [109].

### 2.8. Influence of Fatigue on the Reproductive System

**Figure 4:**
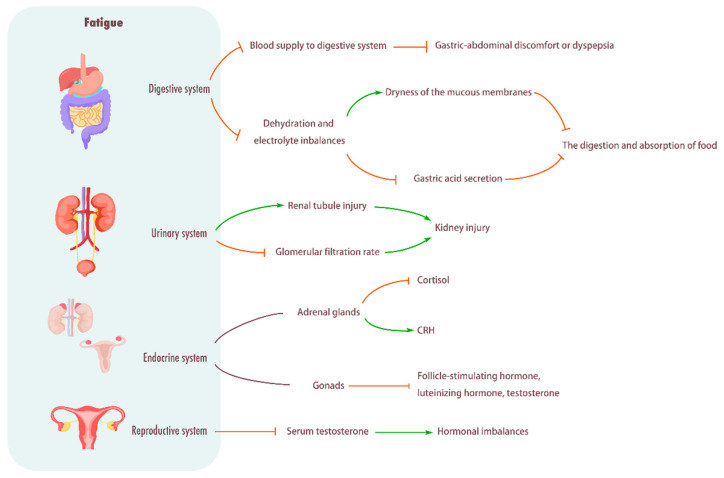
Influence of fatigue on digestive system, urinary system, endocrine system, and reproductive system. Digestive system: Fatigue will give priority to the distribution of blood to the working muscles, reduce the blood supply to the digestive system, weaken the digestive function, and cause stomach and abdomen discomfort or indigestion. In addition, fatigue can also cause dehydration and electrolyte imbalance, dry mucosa, and decreased gastric acid secretion, thus affecting food digestion and absorption. Urinary system: Causes renal tubular damage, decreases glomerular filtration rate, and causes kidney damage. Endocrine system: Fatigue can break the balance of the endocrine system, and then affect the function of the adrenal gland and gonad, resulting in the decrease in cortisol level and the hyperactivity of CRH, as well as the significant decrease in plasma luteinizing hormone, follicle-stimulating hormone and testosterone concentrations. Reproductive system: Fatigue will cause a significant decrease in serum testosterone levels, resulting in hormonal imbalance.

The reproductive system is controlled by hormones secreted by various glands in the body, including the pituitary gland and gonads (testicles in men and ovaries in women). These hormones regulate the development and function of the reproductive organs and the menstrual cycle in women. The reproductive system is essential for the continuation of the species, and it can create new life through sexual reproduction. The effect of fatigue on the reproductive system is mainly manifested in the secretion of sex hormones. As a sex hormone in the body, serum testosterone plays an important role in the physical and mental health of both men and women [110]. It is mainly secreted by the testicles in men and the ovaries and adrenal glands in women, and plays an important role in maintaining the strength and volume of an individual’s muscles, improving exercise capacity, and promoting sexual function. After excessive exercise, the body will develop sports fatigue, which will affect the secretion of serum testosterone. The researchers found that serum testosterone levels in male athletes decreased significantly (from 673 to 303 ng/dL) after marathon and returned to normal levels after adequate rest. Therefore, they believe that marathons cause significant physical stress among athletes, which leads to significant hormonal imbalances and severe cell damage [111] (Figure 4).

## 3. Summary

Based on etiology, fatigue can be categorized into exercise-induced, occupational, and pathological types, each with distinct yet interconnected mechanisms leading to systemic functional decline. Rather than an isolated phenomenon, fatigue represents a multi-level pathological process involving metabolic, structural, and functional alterations across multiple organ systems. Specifically, it manifests as impaired muscle function, dysregulated bone metabolism, and joint instability in the motor system; neuro-metabolic disturbances, neurotransmitter dysregulation, and central/peripheral neural alterations in the nervous system; compromised myocardial energy metabolism and endothelial dysfunction in the cardiovascular system; and functional disruptions in the digestive, urinary, endocrine, and reproductive systems. Notably, immune system dysregulation may play a crucial role in fatigue-related multi-organ dysfunction. The elevation of pro-inflammatory cytokines can exacerbate the increase in muscle protein breakdown, impair synaptic plasticity in the central nervous system, and induce endothelial inflammation, among other effects. Therefore, future research focusing on elucidating the interaction mechanisms between fatigue and the immune system, as well as clarifying the organ-specific phenotypes mediated by immune pathways, will provide a critical theoretical basis for the development of targeted anti-fatigue therapeutic strategies.

## Data Availability

No new data were created or analyzed in this study.
